# Abnormal lateralization of functional connectivity between language and default mode regions in autism

**DOI:** 10.1186/2040-2392-5-8

**Published:** 2014-02-06

**Authors:** Jared A Nielsen, Brandon A Zielinski, P Thomas Fletcher, Andrew L Alexander, Nicholas Lange, Erin D Bigler, Janet E Lainhart, Jeffrey S Anderson

**Affiliations:** 1 Interdepartmental Program in Neuroscience, University of Utah, 20 North 1900 East, Salt Lake City, UT 84132, USA; 2 Department of Psychiatry, University of Utah, 501 Chipeta Way, Salt Lake City, UT 84108, USA; 3 Department of Pediatrics and Neurology, University of Utah and Primary Children’s Medical Center, 295 Chipeta Way, Salt Lake City, UT 84108, USA; 4 School of Computing and Scientific Computing and Imaging Institute, University of Utah, 72 Central Campus Dr, Salt Lake City, UT 84112, USA; 5 Department of Psychiatry, and Waisman Laboratory for Brain Imaging and Behavior, University of Wisconsin, 1500 Highland Avenue, Madison, WI 53705, USA; 6 Department of Medical Physics, University of Wisconsin, 1111 Highland Avenue, Madison, WI 53705, USA; 7 Department of Psychiatry and Biostatistics, Harvard University, 401 Park Drive, Boston, MA 02215, USA; 8 Neurostatistics Laboratory, McLean Hospital, 115 Mill Street, Belmont, MA 02478, USA; 9 Department of Psychology and Neuroscience Center, Brigham Young University, 1001 Kimball Tower, Provo, UT 84602, USA; 10 The Brain Institute of Utah, University of Utah, 36 South Wasatch Drive, Salt Lake City, UT 84112, USA; 11 Department of Bioengineering, University of Utah, 20 S. 2030 E., Salt Lake City, UT 84112, USA; 12 Department of Radiology, 1A71 School of Medicine, University of Utah, Salt Lake City, UT 84132, USA

**Keywords:** brain lateralization, brain asymmetry, autism, autism spectrum disorder, language, functional magnetic resonance imaging, functional connectivity

## Abstract

**Background:**

Lateralization of brain structure and function occurs in typical development, and abnormal lateralization is present in various neuropsychiatric disorders. Autism is characterized by a lack of left lateralization in structure and function of regions involved in language, such as Broca and Wernicke areas.

**Methods:**

Using functional connectivity magnetic resonance imaging from a large publicly available sample (n = 964), we tested whether abnormal functional lateralization in autism exists preferentially in language regions or in a more diffuse pattern across networks of lateralized brain regions.

**Results:**

The autism group exhibited significantly reduced left lateralization in a few connections involving language regions and regions from the default mode network, but results were not significant throughout left- and right-lateralized networks. There is a trend that suggests the lack of left lateralization in a connection involving Wernicke area and the posterior cingulate cortex associates with more severe autism.

**Conclusions:**

Abnormal language lateralization in autism may be due to abnormal language development rather than to a deficit in hemispheric specialization of the entire brain.

## Background

Brain lateralization occurs during typical development [1]. Many reports exist of lateralized brain function underlying cognitive and behavioral processes, such as memory [2] and emotional processing [3]; however, the two most common reports of lateralized brain function are in relation to language and visuospatial processing [4-6]. Most typically developing individuals have significant left lateralization in language regions [7] and right lateralization in attentional regions [4].

Two recent reports describe how lateralized brain function segregates into two broad networks - a right- and left-lateralized network - in typical development [8,9]. The left-lateralized network appears to participate more in intrahemispheric connections, while the right-lateralized network participates in connections between hubs of the network and brain regions in both hemispheres [9]. In one report, the broad networks include 20 lateralization hubs, nine in the left-lateralized network and 11 in the right-lateralized network. The left-lateralized network includes core language regions (Broca and Wernicke areas) and regions of the default mode network (posterior cingulate cortex, medial prefrontal cortex, and lateral temporal parietal junction, among other areas) [8]. The right-lateralized network includes regions from three networks associated with attention to external stimuli: the dorsal and ventral attention networks and the frontoparietal executive network.

Atypical lateralization in brain structure and function is associated with neuropsychiatric conditions and developmental disorders such as autism, schizophrenia, and specific language impairment [10-16]. More specifically, autism is associated with reduced left lateralization or reversed lateralization of brain structure and function in core language regions and the white matter tracts that connect them. Abnormal brain lateralization in autism has been measured by multiple techniques, including magnetic resonance imaging (MRI) [10,17,18], functional MRI [11,19,20], diffusion imaging [14,15,21], positron emission tomography [22-24], and electroencephalography [25-27]. It has been reported throughout the lifespan in infancy and childhood [20,24-29], adolescence [20], and adulthood [22,23]. Lateralization of brain function correlates with language ability in individuals with autism [25].

In contrast with reports of abnormal lateralization restricted to language-related regions, autism is more generally characterized by connectivity abnormalities across many large-scale brain networks. Abnormal connectivity observations in autism have been made using both functional and structural connectivity analyses [30,31]. Long-range connections between distributed connections are underconnected in autism [32], although reports of overconnectivity also exist [33]. The abnormal connections are found in default mode, motor, social, language, face processing, and salience networks, among others [30,34-48]. These core findings have been confirmed in a multisite dataset with over 1,000 subjects [49,50]. These studies suggest the pathophysiology of autism includes widespread deficits across structural and functional networks, rather than deficits confined to a single brain region.

The majority of reports on brain lateralization in autism focus on abnormal lateralization in language-related regions. It is unclear whether this is because language is typically associated with lateralized brain function and language impairment is a core feature of autism, or because abnormal lateralization in autism is truly most pronounced in language-related regions. To answer this question, Cardinale and colleagues (2013) characterized whether functional lateralization abnormalities in autism existed outside of language-specific regions. They found diffuse differences across many different functional networks [51]. These widespread differences in functional lateralization existed in a small sample of children and adolescents (n = 20 for both groups), using independent component analysis to identify the functional networks. In light of Cardinale and colleagues’ findings and the widespread connectivity differences in autism, we hypothesized that lateralization abnormalities would be present across multiple networks.

In the present study, we investigated the 20 lateralization hubs that form the two lateralized networks reported previously [8], using a multisite dataset with over 1,000 participants. We studied whether the lateralization of brain function differs between autism and typical development in a diffuse, network-wide manner or within isolated language-related brain regions. We also investigated whether lateralization of brain function correlates with clinical severity, age, and handedness.

## Methods

### Subject sample

The Autism Brain Imaging Data Exchange (ABIDE) consists of 1112 datasets comprised of 539 autism and 573 typically developing individuals [49]. Each dataset consists of one or more resting functional MRI acquisitions and a volumetric magnetization-prepared rapid acquisition with gradient echo (MPRAGE) image. All data are fully anonymized in accordance with Health Insurance Portability and Accountability Act (HIPAA) guidelines, with analyses performed in accordance with pre-approved procedures by the University of Utah Institutional Review Board. All images were obtained with informed consent according to procedures established by human subjects research boards at each participating institution. Details of acquisition, informed consent, and site-specific protocols are available at http://fcon_1000.projects.nitrc.org/indi/abide/.

The majority of the analyses were done on 964 (517 typically developing subjects and 447 subjects with autism from 16 sites and 19 datasets because three sites had multiple datasets) of the 1,112 ABIDE subjects who met the following inclusion criteria: 1) successful normalization to Montreal Neurological Institute (MNI) space of MPRAGE verified by manual visual inspection, 2) co-registration of blood-oxygen-level dependent (BOLD) and MPRAGE images, 3) segmentation of MPRAGE image, 4) full brain coverage from MNI z > -35 to z <70 on all BOLD images, and 5) the subject must be a part of a site where at least 20 subjects met inclusion criteria 1 to 4. We also did secondary analyses using more strict inclusion criteria (see footnotes B to H in Table 1) applied separately or in tandem with other inclusion criteria. The more strict inclusion criteria required, first, that a subject have at least 50% of his or her resting state BOLD volumes remaining after motion scrubbing. Second, some of the ABIDE data for the typically developing controls were included in the 1000 Functional Connectomes (http://fcon_1000.projects.nitrc.org/) and/or ADHD-200 samples (http://fcon_1000.projects.nitrc.org/indi/adhd200/). The 1000 Functional Connectomes and ADHD-200 datasets were used as the basis for the 20 lateralization hubs interrogated in the present study [8]. We were not able to determine which subjects were present in both the ABIDE sample and the 1000 Functional Connectomes or ADHD-200 samples due to anonymous submission of data to the publicly available samples. Therefore, we excluded sites where there was possible overlap in samples. Third, we included only right-handed subjects and excluded left-handed, mixed-handed, and ambidextrous subjects. Fourth, we included only male subjects. Fifth, we included subjects diagnosed with autism and excluded subjects diagnosed with Asperger Syndrome or Pervasive Developmental Disorder-Not Otherwise Specified (PDD-NOS). Finally, we matched the groups based on verbal intelligence quotient (IQ). In order to do so, we included subjects with autism who had a verbal IQ between 80 and 130 and typically developing subjects who had a verbal IQ between 70 and 120.

**Table 1:** Group differences in lateralization for various subject inclusion criteria

**Inclusioncriteria**	**Total n (Autism n)**	**Region of interest 1**	**Region of interest 2**	***t***	***P***
**A**	964 (447)	Posterior cingulate	Wernicke	3.37	7.7 × 10^-4^
		Posterior cingulate	Broca	3.04	2.4 × 10^-3^
		Temporoparietal junction	Wernicke	3.63	2.9 × 10^-4^
**B**	831 (362)	Posterior cingulate	Wernicke	3.39	7.2 × 10^-4^
		Posterior cingulate	Lateral premotor	2.93	3.5 × 10^-3^
		Temporoparietal junction	Wernicke	3.66	2.7 × 10^-4^
**C**	765 (447)	Posterior cingulate	Wernicke	3.69	2.4 × 10^-4^
		Posterior cingulate	Broca	3.52	4.6 × 10^-4^
		Posterior cingulate	Lateral premotor	3.63	3.0 × 10^-4^
		Posterior cingulate	Left supplementary motor area	2.74	6.3 × 10^-3^
		Temporoparietal junction	Wernicke	3.78	1.6 × 10^-4^
		Temporoparietal junction	Left supplementary motor area	2.87	4.2 × 10^-3^
**D**	645 (362)	Posterior cingulate	Wernicke	3.83	1.4 × 10^-4^
		Posterior cingulate	Lateral premotor	3.79	1.7 × 10^-4^
		Temporoparietal junction	Wernicke	3.71	2.3 × 10^-4^
**E**	850 (378)	Temporoparietal junction	Wernicke	3.33	8.9 × 10^-4^
**F**	822 (396)	Posterior cingulate	Wernicke	3.36	8.3 × 10^-4^
		Temporoparietal junction	Wernicke	3.30	1.0 × 10^-3^
**G**	765 (280)	Posterior cingulate	Wernicke	3.30	1.0 × 10^-3^
		Posterior cingulate	Broca	2.93	3.5 × 10^-3^
		Temporoparietal junction	Wernicke	3.04	2.4 × 10^-3^
		Medial prefrontal	Wernicke	2.74	6.2 × 10^-3^
		Posterior cingulate	Lateral premotor	3.36	8.3 × 10^-4^
**H**	610 (309)	Temporoparietal junction	Wernicke	3.22	1.3 × 10^-3^

A: Met all preprocessing criteria (described in Methods section) and part of site with >20 subjects.

B: Criteria A + subject has >50% resting state BOLD volumes after motion scrubbing.

C: Criteria A + subject not included in 1000 Functional Connectomes or ADHD200 datasets.

D: Criteria A + B + C.

E: Criteria A + right-handed subjects only.

F: Criteria A + male subjects only.

G: Criteria A + autism only (that is, removed individuals with Asperger’s syndrome and Pervasive Developmental Disorder- Not Otherwise Specified (PDD-NOS). H: Criteria A + matched groups on verbal IQ (80 < autism verbal IQ <130 and 70 < control verbal IQ <120).

Each site followed different criteria for diagnosing patients with autism or ascertaining typical development; however, the majority of the sites used the Autism Diagnostic Observation Schedule [52] and Autism Diagnostic Interview-Revised [53]. Specific diagnostic criteria for each site can be found at fcon_1000.projects.nitrc.org/indi/abide/index.html. Subject demographics for individuals satisfying inclusion criteria are shown in Table 2. Six different testing batteries were used to calculate verbal IQ and performance IQ, respectively. Specific IQ testing batteries and other behavioral measures for each site can be found at fcon_1000.projects.nitrc.org/indi/abide/index.html. In the case that no categorical measure of handedness (that is, right-handed, left-handed, or ambidextrous) was reported but a quantitative measure (that is, -100 to +100 with -100 representing strongly left-handed and +100 representing strongly right-handed) was reported, positive values from the quantitative measure were converted to right-handed, negative values to left-handed, and a value of zero to ambidextrous. Fifteen subjects lacked both a quantitative and categorical measurement of handedness.

**Table 2:** Subjects included from the ABIDE sample with demographic information

		**Age**	**ADOS total**	**Handedness (left, right, ambidextrous or mixed)**	**Handedness(-100 to +100)**	**Verbal IQ**	**Performance IQ**
**Control**		(426 M, 91 F)	32	(472 R, 34 L, 3 A)	184	413	425
**Autism**		(396 M, 51 F)	316	(378 R, 58 L, 4 A)	164	367	371
**Control mean +/- s.d.**		16.9 +/- 7.56	1.25 +/- 1.37	N/A	67.4 +/- 39.0	112 +/- 13.3	108 +/- 13.3
	**(Control range)**	(6.47 - 56.2)	(0 to 4)	N/A	(-100 to +100)	(67 to 147)	(67 to 155)
**Autism mean +/- s.d.**		16.6 +/- 8.1	11.9 +/- 3.81	N/A	51.8 +/- 54.5	105 +/- 17.4	106 +/- 17.2
	**(Autism range)**	(7 to 64)	(2 to 22)	N/A	(-100 to +100)	(50 to 149)	(59 to 157)

A, Ambidextrous

ADOS, Autism Diagnostic Observation Schedule

IQ, Intelligence quotient

L, Left

R, Right

s.d., Standard deviation

### BOLD preprocessing

Preprocessing was performed in MATLAB (Mathworks, Natick, MA, USA) using SPM8 (Wellcome Trust, London, UK) software. The following sequence of preprocessing steps was performed:

1) Slice timing correction

2) Realign and reslice correction of motion for each volume relative to initial volume

3) Co-registration of BOLD images to MPRAGE anatomic sequence

4) Normalization of MPRAGE to MNI template brain, with normalization transformation also being applied to co-registered BOLD images

5) Segmentation of gray matter, white matter (WM), and cerebrospinal fluid (CSF) components of MPRAGE image (thorough clean)

6) Extraction of mean time courses from the restriction masks applied to BOLD images from regions of interest (ROIs) consisting of:

a. CSF segmented mask with bounding box -35 < x < 35, -60 < y < 30, 0 < z < 30

a. White matter segmented mask overlapping with 10 mm radii spheres centered at

x = -27, y = -7, z = 30, x = 27, y = -7, z = 30

a. Mask of scalp and facial soft tissues [54]

7) Voxelwise bandpass filter (0.001 to 0.1 Hz) and linear detrend, performed concurrently with step 8.

8) Voxelwise regression using glmfit.m (MATLAB Statistics Toolbox, Mathworks, Natick, MA, USA) software of CSF, WM, Soft tissue, and 6 motion parameters from realignment step from time series of each voxel of BOLD images

9) Motion scrubbing [55] of first, framewise displacement, and second, the root mean squared change in BOLD signal from volume to volume (DVARS) with removal of volumes before and after a root-mean-square displacement of >0.2 for either parameter and concatenation of remaining volumes. The subjects with autism move more in the scanner compared to the typically developing subjects both before (autism motion = 0.15 +/- 0.14 mm; typically developing motion = 0.11 +/- 0.08 mm; *t*(962) = 5.68, *P* = 1.8 × 10^-8^) and after scrubbing (autism motion = 0.08 +/- 0.02 mm; typically developing motion = 0.07 +/- 0.02 mm; *t*(962) = 5.56, *P* = 3.5 × 10^-8^). The group with autism retained 73.8 +/- 25.8% of the BOLD volumes after scrubbing, whereas the typically developing group retained 82.3 +/- 22.1%. However, we do not believe the differences in motion affect the overall results because we compared one hemisphere’s connectivity with the other hemisphere’s connectivity within a single subject before comparing across groups. Unless motion alters connectivity differently across hemispheres, the functional lateralization metric should not be affected.

10) No spatial smoothing was performed to avoid contaminating the signal near the midsagittal plane. The global mean signal and gray matter time courses were not regressed from voxelwise data [54,56-58].

### Region of interest analysis

From preprocessed BOLD images for each subject, mean time course was extracted from 7,266 gray matter ROIs. These ROIs form a lattice covering the grey.nii image (SPM8) from z = -35 to z = 70 at 5-mm resolution, with MNI coordinates of centroids previously reported [34]. The ROIs averaged 4.9 +/- 1.3 standard deviation voxels in size for 3 mm isotropic voxels. A 7,266 × 7,266 matrix of Fisher-transformed Pearson correlation coefficients was obtained for each subject from the ROI time courses representing an association matrix of functional connectivity in each subject between all pairs of ROIs. Each pair of ROIs is termed a ‘connection’ for the present analysis.

### Functional lateralization metric

Functional correlation was obtained as the Fisher-transformed Pearson correlation coefficient between each pair of the 7,266 ROIs within the same hemisphere. We only analyzed connections within a single hemisphere and the opposite hemisphere homologues because of ambiguity of ‘lateralization’ of a cross-hemisphere connection. Preprocessed images were inverted across the midsagittal plane, and analogous Fisher-transformed correlation coefficients were obtained between each pair of the same ROIs on the flipped images. Functional lateralization index was defined as the difference (unflipped - flipped) between Fisher-transformed correlation coefficients.

**Figure 1:**
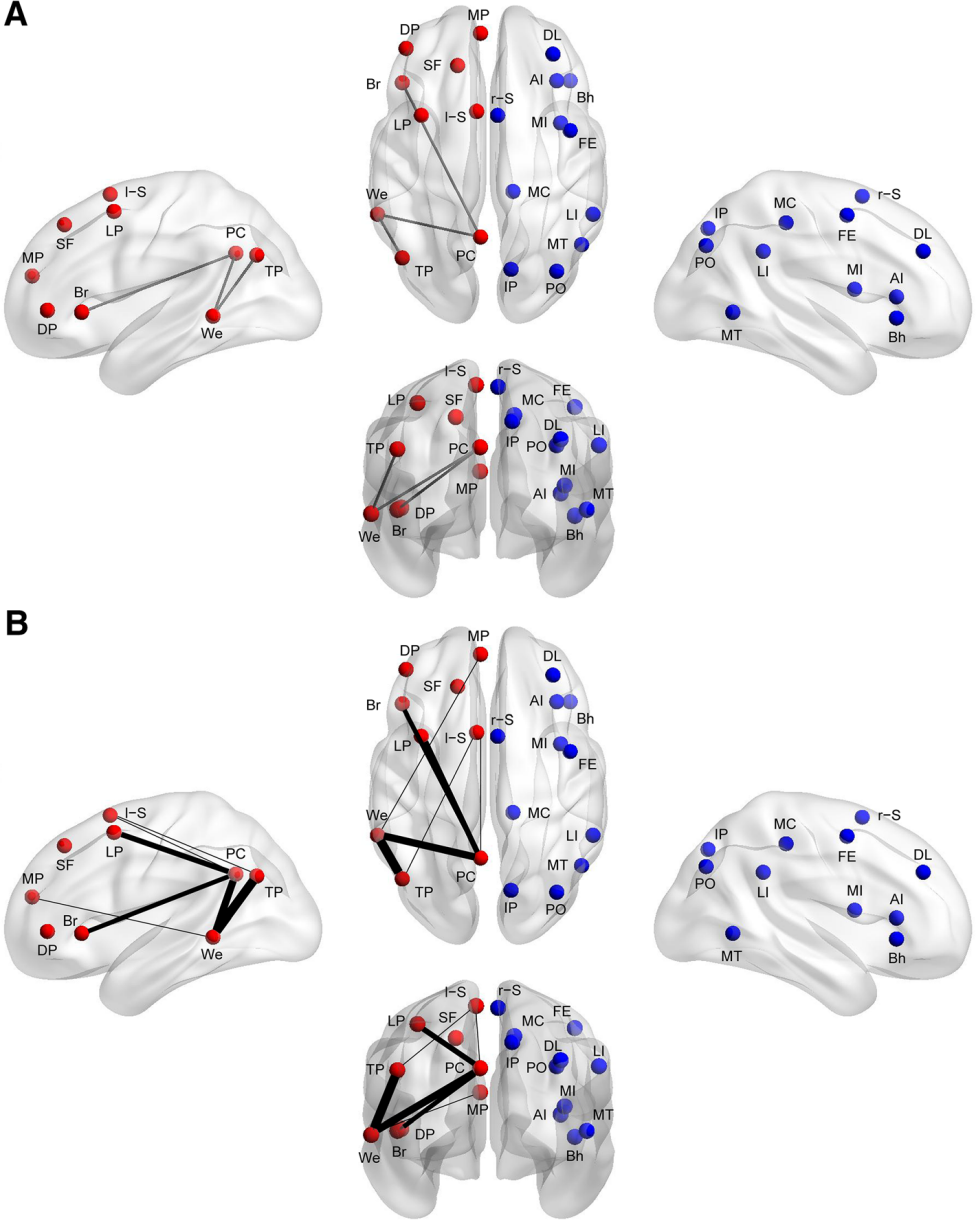
**Lateralized hub locations and abnormally lateralized connections.** The left- (red) and right-lateralized (blue) brain regions that participate in the most left- and right-lateralized connections, as determined in a separate sample of 1,011 typically developing subjects, are displayed on rendered brain images. **A)** Three connections (black lines) are less left-lateralized in the autism group compared to the typically developing group when all 964 subjects are included in the analysis. **B)** All of the connections with an false discovery rate (FDR)-corrected group difference for at least one of the eight criteria in Table 1 are displayed. The lines between the regions of interest (ROIs) are weighted according to the number of criteria that the connections met FDR-corrected significance. Abbreviations: AI, anterior insula; Bh, Broca homologue; Br, Broca area; DL, dorsolateral prefrontal cortex; DP, inferior dorsolateral prefrontal cortex; FE, frontal eye fields; IP, superior medial intraparietal sulcus; LI, lateral intraparietal sulcus; LP, lateral premotor cortex; l-S, left supplementary motor area; MC, mid cingulate cortex; MI, mid insula; MP, medial prefrontal cortex; MT, middle temporal area; PC, posterior cingulate cortex; PO, parietooccipital cortex; r-S, right supplementary motor area; SF, medial superior frontal cortex; TP, temporoparietal junction; We, Wernicke area.

In a previous study of typical development, 20 cortical regions were identified as lateralization hubs, or brain regions involved in the most functionally lateralized connections (Figure 1) [8]. The 20 lateralization hubs were a subset of 7,266 ROIs described above and comprised 9 left-hemispheric regions and 11 right-hemispheric regions. All analyses in the present study focused on connections between the 20 lateralization hubs. MNI coordinates of the 20 lateralization hubs and detailed information on the methods for identifying the lateralization hubs have been previously reported [8]. In order to determine the signal quality for the 20 lateralization hubs, the signal-to-noise ratio (SNR) was calculated by averaging the BOLD signal intensity across the entire resting state scan (using the slice-timing corrected, motion corrected, and normalized images in step 4 from the ‘BOLD preprocessing’ section above) for each hub separately and then dividing by the signal’s standard deviation. The mean SNR for the 20 lateralization hubs across the 964 subjects is moderate to high and ranges between 72 and 110 (similar to SNR reported by Yeo and colleagues [59]).

### Statistical analyses

**Figure 2:**
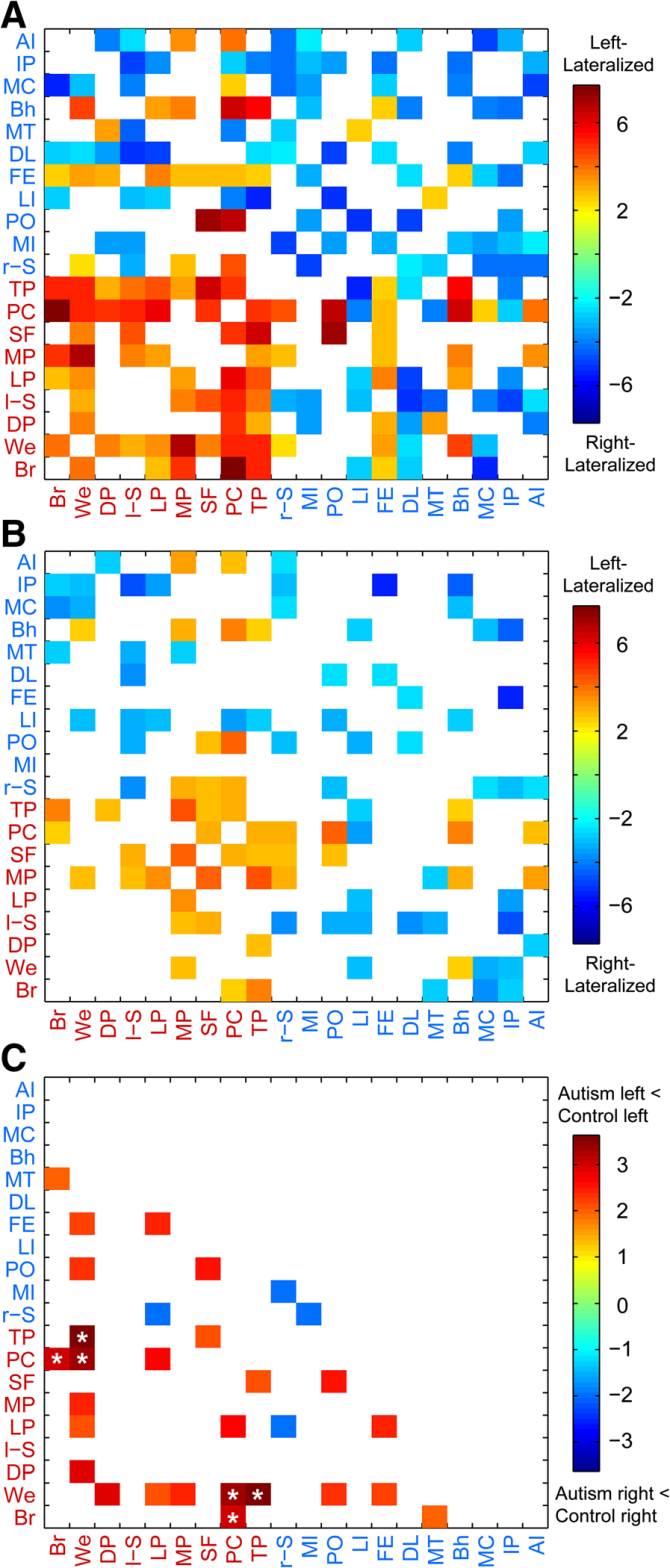
**Group lateralization patterns.** The lateralization patterns of the connections involving the 20 lateralized hubs displayed in the typically developing group **(A)**, autism group **(B)**, and group differences **(C)**. The colored connections (that is, squares of the plot) represent a group difference of *P* <0.05 and colored connections with asterisk represent a group difference that survives multiple comparisons correction using a false discovery rate of *q* <0.05. Abbreviations: AI, anterior insula; Bh, Broca homologue; Br, Broca area; DL, dorsolateral prefrontal cortex; DP, inferior dorsolateral prefrontal cortex; FE, frontal eye fields; IP, superior medial intraparietal sulcus; LI, lateral intraparietal sulcus; LP, lateral premotor cortex; l-S, left supplementary motor area; MC, mid cingulate cortex; MI, mid insula; MP, medial prefrontal cortex; MT, middle temporal area; PC, posterior cingulate cortex; PO, parietooccipital cortex; r-S, right supplementary motor area; SF, medial superior frontal cortex; TP, temporoparietal junction; We, Wernicke area.

**Figure 3:**
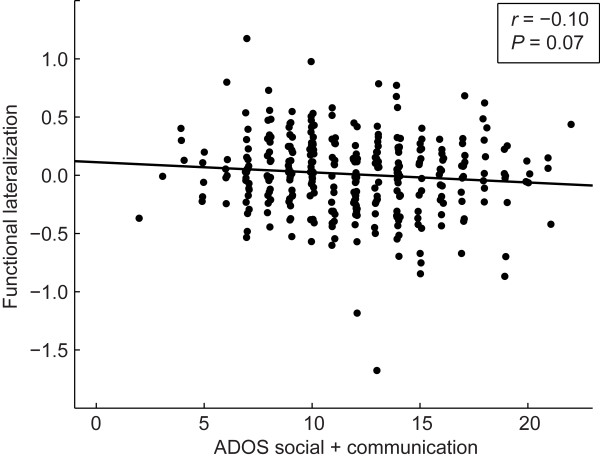
**Relationship between functional lateralization and autism severity.** The left lateralization of the functional connection involving Wernicke area and posterior cingulate cortex shares a trend toward a negative correlation with autism severity (*r* = -0.10, *P* = 0.07), as calculated by adding the Autism Diagnostic Observation Schedule (ADOS) social and communication domains’ total scores for each subject with autism.

All statistical analyses were performed in MATLAB using MATLAB’s statistical toolbox. Each lateralization hub’s pattern of lateralization with other hubs in the ipsilateral hemisphere of the cerebral cortex was determined separately for the typically developing group and the autism group by performing one-sample *t*-tests on the functional connections involving the cortical hub as the seed and the other ipsilateral hubs (Figure 2). We corrected for multiple comparisons using acceptable false discovery rate of *q* <0.05. In the case where connections involved contralateral hubs (that is, a connection involving both a left-lateralized hub and a right-lateralized hub), the right-lateralized hub was flipped across the midsagittal plane and the test of lateralization was made as if both hubs were in the left hemisphere. This was done to allow for feasible interpretations on lateralization between the left-hemispheric and right-hemispheric hubs. To test for group differences in lateralization of intrinsic connectivity, two-sample *t*-tests were applied on the set of ipsilateral connections involving the 20 lateralization hubs (36 left-lateralized connections and 55 right-lateralized connections; Figures 1 and 2). We again corrected for multiple comparisons using acceptable false discovery rate of *q* <0.05. We also used different inclusion criteria for the subjects when testing group differences in lateralization of the 91 lateralized connections (Table 1). To test for differences in the degree of lateralization, we first found each subject’s average functional lateralization for the following three groups of connections: 1) 15 left-lateralized connections involving language regions (that is, Broca and/or Wernicke area), 2) 21 left-lateralized connections not involving language regions (that is, the other seven left-lateralized hubs), and 3) 55 right-lateralized connections. Then, we used paired-sample *t*-tests for the two groups separately comparing the mean functional lateralization for the three groups of connections. To test for the effect of clinical severity, age, and quantitative handedness, Pearson correlation coefficients (or Spearman rank correlation coefficients for age and handedness due to non-normality in residuals) were calculated across all participants for the three connections with abnormal lateralization when comparing the typically developing group to the autism group (Figure 3).

## Results

We investigated the lateralization patterns among the lateralization hubs of the left- and right-lateralized networks in typical development and autism, and then compared the lateralization patterns of the two groups. In the typically developing group, strong lateralization existed between the hubs of the left- and right-lateralized networks, respectively (Figure 2A). The hubs in the right hemisphere are part of right-lateralized connections that form a right-lateralized network. The hubs in the left hemisphere are part of left-lateralized connections that form a left-lateralized network. In the autism group, lateralization between the hubs also existed, although not as strongly as in the typically developing group (Figure 2B). When comparing the two groups, the majority of the differences existed in connections involving specific left-lateralized hubs (Figure 1 and Figure 2C). Only three of the connections survived multiple comparisons correction using a false discovery rate of *q* <0.05. The three connections were in the left-lateralized network: the Wernicke area to the posterior cingulate cortex; the Wernicke area to the temporoparietal junction; and the Broca area to the posterior cingulate cortex. All three either lacked left lateralization or had greatly diminished left lateralization in the autism group compared to the typically developing group (Wernicke-posterior cingulate: *t*(961) = 3.36, *P* = 0.0008; Wernicke-temporoparietal: *t*(962) = 3.30, *P* = 0.001; Broca-posterior cingulate: *t*(960) = 3.04, *P* = 0.002).

We also repeated the analyses that identified the group differences in lateralized functional connections, using seven additional inclusion criteria to determine which subjects would be included in the analysis (Table 1). The connections that were most consistently abnormal in the autism group involved the Wernicke area and the posterior cingulate cortex (abnormal in criteria A to D, F, and G of Table 1) and the connection involving Wernicke area and temporoparietal junction (abnormal in criteria A to H of Table 1). Five other connections were abnormal in at least one of the seven inclusion criteria analyses, all involving core language regions and default mode regions in the left-lateralized network (Figure 1B).

Next, we compared the degree of lateralization for three groups of connections (that is, left-lateralized connections involving language regions, left-lateralized connections not involving language regions, and right-lateralized connections) in typical development and autism separately. In typical development, the left-lateralized connections (both connections involving language regions and connections not involving language regions) were more left-lateralized than the right-lateralized connections were right-lateralized (Language: *t*(514) = 3.97, *P =* 0.00008; Non-language: *t*(514) = 2.77, *P =* 0.006). The left-lateralized connections involving language regions were slightly more left-lateralized than the left-lateralized connections not involving language regions, although the difference was not significant (*t*(514) = 1.84, *P =* 0.07). In contrast, the autism group’s left-lateralized connections not involving language regions were more left-lateralized than the left-lateralized connections involving language regions (*t*(440) = 2.90, *P =* 0.004). Also, the left-lateralized connections involving language regions were as left-lateralized as the right-lateralized connections were right lateralized (*t*(440) = 0.39, *P =* 0.70); whereas, the left-lateralized connections not involving language regions were more left-lateralized than the right-lateralized connections were right-lateralized (*t*(440) = 3.35, *P =* 0.0009).

Finally, we investigated the relationship between lateralization in the three abnormal connections and autism severity, age, and handedness. We observed a trend toward less left lateralization in the connection between Wernicke area and the posterior cingulate cortex with increased autism severity (*r*(314) = -0.10, *P =* 0.07; Figure 3). If control subjects are included for whom ADOS scores were available, these results are more significant (*r*(346) = -0.13, *P =* 0.017). No significant relationships between lateralization and age or lateralization and handedness were found in either group.

## Discussion

In this study, we tested brain lateralization in autism using functional connectivity MRI and found that abnormal lateralization of functional connectivity during rest in autism is most pronounced in specific left-lateralized connections that involve language regions (that is, Broca area and Wernicke area) and regions of the default mode network (that is, temporoparietal junction and posterior cingulate cortex), rather than diffusely affecting either the left- or right-lateralized functional networks. We also replicated previous results in the typically developing group that two interconnected lateralized networks exist in the brain, one in the left hemisphere, and one in the right hemisphere, with the left-lateralized network involving language and default mode regions, and the right-lateralized network involving brain attentional regions [8].

Cardinale and colleagues found that abnormal lateralization in autism existed across many intrinsic networks, including primary sensory and higher-level association networks [51]. We, too, found either a lack of left lateralization or greater right lateralization in the autism group; however, the regions or networks involved in abnormal lateralization differed. Rather than finding abnormalities throughout a number of networks as Cardinale and colleagues did, we only found significant differences after multiple comparison corrections in a handful of connections involving language regions and regions of the default mode network. Cardinale and colleagues did find lateralization in the default mode network in some of their supplemental analyses; however, they did not directly test lateralization between language regions and default mode regions.

The inconsistent results may reflect differences in the sample age, sample size, number of data acquisition sites, and/or data analysis methods. In Cardinale *et al*., aggregate network measures were studied that pooled information across many ROI’s, whereas the present study used a more spatially localized approach tailored to study individual ‘connections’ between discrete brain regions. It is possible that subtle or subthreshold differences in lateralization in regions of the brain distinct from core language hubs, when pooled across entire functional networks, yield significant lateralization differences that may not survive rigorous statistical testing when evaluating small discrete ROI’s. In fact, we find this likely given the results of Figure 2, in which many more connections, including some that are not associated with language regions, exhibit decreased lateralization with *P* <0.05. Virtually all of these show decreased lateralization in autism. Given the consistent direction of the effect, it seems probable that when pooled together, these connections may result in more widespread network differences in lateralization. Nevertheless, our results suggest the effect is much stronger in core language and default mode regions and our approach allows a more spatially localized assessment of effect size.

Neither our study nor the Cardinale *et al*. study found a relationship between abnormal lateralization of intrinsic networks and social or communication impairments that survived multiple comparisons [51]. This corresponds with variable relationships found between abnormal brain lateralization and functional connectivity in general. In individuals with autism, reduced functional connectivity within the default mode network relates to more social and communication impairments [34,41,45-47]; however, other studies found no relationship between activation patterns or abnormal lateralization and autism severity or language ability [9,19].

The abnormal lateralization of connections involving regions of the default mode network and core language regions may represent an overall lack of specialization in brain regions that process language and social stimuli. Regions of the default mode network are involved in tasks that require language (for example, internal narrative and autobiographical memory) and theory of mind or understanding of another’s mental state [60-62]. The temporoparietal junction and posterior cingulate cortex participate in the same component as core language regions during a language task [63]. The temporoparietal junction participates in both semantic tasks and deactivates during cognitively taxing tasks (that is, has default mode characteristics) [64]. The posterior cingulate cortex is more active in congruent and coherent language compared to incongruent or incoherent language [65,66]. The right inferior frontal gyrus is more active in autism compared to typical development during a language task, implying abnormal lateralization in a core language region that may have implications in its relationship with other brain regions (for example, as we found with the connection between Broca area and posterior cingulate cortex) [67]. Together these observations suggest the abnormal lateralization between core language regions and default mode regions could account for some of the communication and social deficits experienced by individuals with autism. This possibility is also supported by findings that abnormal lateralization in language regions are correlated with decreased function on standardized testing [9].

Reports of abnormal functional lateralization in specific language impairment correspond with previous reports in autism and the present study. Individuals with specific language impairment have less left-lateralized activation in Broca and Wernicke areas during speech tasks [16,68]. Individuals with developmental dyslexia also have less lateralization across the left hemisphere, as assessed by functional transcranial Doppler ultrasound [69]. One study of note, however, found somewhat different results [9]. It compared individuals with a history of specific language impairment but lacked a current diagnosis, individuals with a current diagnosis of specific language impairment, individuals with autism, and typically developing individuals. Over 80% of the individuals with a current diagnosis of specific language impairment showed right lateralization or bilateral activation during a language task, whereas over 90% of the individuals from the other three groups showed left lateralization. From this study, it appears abnormal lateralization is even more specific to individuals with a current diagnosis of specific language impairment.

The observation that abnormal functional lateralization in autism is most pronounced in connections between core language regions constrains hypotheses of developmental pathophysiology in autism. Our analysis suggests that abnormal language lateralization in autism may be due to abnormal language development rather than a deficit in hemispheric specialization of the entire brain, and would be more consistent with a search for mechanisms involving brain substrates for language acquisition rather than earlier potential mechanisms where hemispheric asymmetries emerge. This constraint is also supported by multimodal observations from DTI, functional MRI, structural MRI, and electrophysiologic studies that have all identified specific deficits in language-related lateralization but not differences in lateralization in other cognitive subsystems.

While the large sample size of the ABIDE dataset can be a tremendous advantage for improving statistical power and external generalizability of the results, it can also be a liability. The individual sites differ in many important data acquisition variables including inclusion criteria, demographics, pulse sequence, scanner type, and length of scan. Most of the included scans were very short, less than 10 minutes duration per subject. It is possible that the heterogeneity of the dataset may limit sensitivity for detecting small changes, and that in a more homogenous data sample, additional differences in lateralization would be found.

An additional limitation is that we did not attempt a discovery of all lateralization differences in an attempt to control the multiple comparison problem that would arise, but instead looked for lateralization differences only between a set of 20 regions that were previously identified as being hubs of lateralized networks in a control population (different from the control subjects used here). It is possible that systematic differences in lateralization are present in brain regions that are not necessarily hubs of lateralization networks in the brain, and which we could not detect. It is also possible that control and autism groups differ in precise spatial coordinates of some lateralization hubs, which we would not be able to detect.

## Conclusions

Brain lateralization occurs in typical development and is abnormal in autism. As has been shown in multiple reports, left lateralization of core language regions in autism is diminished. In addition to core language regions, we have shown that the synchronization between core language regions and default mode regions lacks left-sided lateralization in autism. Also, there is a trend toward abnormal lateralization correlating with more severe communication and social deficits. These abnormalities represent differences that persist from childhood throughout adulthood, in at least a subgroup of individuals with autism, and suggest a lack of specialization.

## Abbreviations

ABIDE: Autism Brain Imaging Data Exchange; ADOS: Autism Diagnostic Observation Schedule; BOLD: blood-oxygen-level dependent; CSF: cerebrospinal fluid; DTI: diffusion tensor imaging; DVARS: root mean squared change in BOLD signal from volume to volume; HIPAA: Health Insurance Portability and Accountability Act; IQ: intelligence quotient; MNI: Montreal Neurological Institute; MPRAGE: magnetization-prepared rapid acquisition with gradient echo; MRI: magnetic resonance imaging; PDD-NOS: pervasive developmental disorder-not otherwise specified; ROI: region of interest; SNR: signal-to-noise ratio; WM: white matter.

## Competing interests

The authors declare that they have no competing interests.

## Authors’ contributions

JN participated in the design of the study, performed the analyses, and wrote the manuscript. BZ helped in acquiring the data, participated in the design of the study, and helped to draft the manuscript. PF helped in acquiring the data, participated in the design of the study, and helped to draft the manuscript. AA helped in acquiring the data, participated in the design of the study, and helped to draft the manuscript. NL helped in acquiring the data, participated in the design of the study, and helped to draft the manuscript. EB helped in acquiring the data, participated in the design of the study, and helped to draft the manuscript. JL helped in acquiring the data, participated in the design of the study, and helped to draft the manuscript. JA participated in the design of the study, performed the analyses, and wrote the manuscript. All authors read and approved the final manuscript.
